# A case of a patient infected with a hepatitis C virus genotype 3a multidrug resistant variant in Pakistan

**DOI:** 10.1186/s40249-018-0386-7

**Published:** 2018-02-11

**Authors:** Asad Zia, Muhammad Ali, Hafsa Aziz, Muhammad Zia, Zabta Khan Shinwari, Abida Raza

**Affiliations:** 1 Department of Biotechnology, Quaid-i-Azam University Islamabad, Islamabad, Pakistan; 2 Nuclear Oncology and Radiotherapy Institute (NORI), Islamabad, Pakistan; 3 National Institute of Lasers and Optronics (NILOP), Nanomedicine Research Labs, Islamabad, Pakistan

## Abstract

**Background:**

Approximately 10 million people in Pakistan are infected with the hepatitis C virus (HCV). Most patients develop chronic hepatitis, with rare cases of spontaneous clearance. However, little is known about multidrug resistant viral variants in Pakistan.

**Findings:**

This case study describes a 47-year-old male diagnosed with chronic HCV genotype 3a infection in 2003. After an initial diagnosis of viral infection, the patient remained treatment naïve for 5 years. He received two therapy cycles of interferon (IFN) plus ribavirin (RBV) in 2007 and 2010, however, he was non-responsive to the therapy. The patient then received an additional two treatment cycles of pegylated IFN α-2b plus RBV (in 2011 and 2013); he was still non-responsive. In 2016, the patient underwent sofosbuvir plus RBV combination therapy, however, the sustained virological response was still not achieved. The host genetic factor was found to be heterozygous guanine and thymine (GT) and cytosine and thymine (CT) genotypes of rs8099917 and rs12979860 polymorphism of IL28B, respectively. Phylogenetic analysis suggests that the resistant variant belong to an out-group and may require triple therapy.

**Conclusions:**

This is the first case that reports on a HCV-infected individual who was a non-responder to multiple IFN therapies in Pakistan. Further studies are needed to understand multidrug-resistant HCV variants in the Pakistani population.

**Electronic supplementary material:**

The online version of this article (10.1186/s40249-018-0386-7) contains supplementary material, which is available to authorized users.

## Multilingual abstracts

Please see additional file 1 for translations of the abstract into the five official working languages of the United Nations.

## Background

The hepatitis C virus (HCV) is a single-stranded positive ribonucleic acid (RNA) virus with a high genetic variability rate. A single infected HCV patient can have a mixture of closely related viral genomes, either as quasispecies or separate groups referred to as genotypes [1]. Genotypes strongly affect outcomes of the standard interferon (IFN) treatment regimen [2, 3]. Different studies have reported that close relationships exist between genotyping, disease behaviour, and response to IFN treatment [4–7]. Patients infected with HCV genotypes 2 and 3 respond more efficiently to IFN-based therapies than those infected with a genotype 1 infection [8]. Sustained virological response (SVR) rates to standard IFN plus ribavirin (RBV) treatment regimens in the HCV-positive population of Pakistan have been reported to be between 27.8% and 62% [9]. Higher SVR rates have been reported in relation to pegylated interferon (PEG-IFN) in HCV-2a infected patients as compared to patients infected with HCV-3a [10–12]. Recently, more effective oral treatment options have been introduced with reported SVR rates of about 90% (sometimes > 90%) and minimal adverse effects.

Sofosbuvir (SOF) is one of the direct-acting antivirals (DAAs) approved by the U.S. Food and Drug Administration (US-FDA). It was approved on December 6, 2013 [13, 14]. In treatment-naïve patients infected with genotype 1, phase 2 trials of 400 mg/day of PEG-IFN, RBV, and SOF for 12 or 24 weeks resulted in SVRs of 87–92% [15, 16].

Although SOF has been available in Pakistan at a subsidized price since December 2014, there is limited literature on treatment response rates [17, 18]. This paper reports on a HCV-infected patient who was non-responsive to multiple combination antiviral therapies.

## Case presentation

A 47-year-old male with a body weight of 84 kg and height of 1.65 m living in underprivileged part of Islamabad has a history of generalized pain, fatigue, and fever. He was diagnosed with chronic HCV (3a genotype) infection in 2003. The patient’s medical history was not significant except for dental surgery and few surgical stitches. He remained treatment naïve for 5 years. In 2007, he received the first IFN (100 mg/week) plus RBV (400 mg/day) combination therapy and was on this treatment for 6 months. However, SVR was not achieved. He remained without treatment for the next 2 years (2008–2009). In 2010, he again underwent the same combination therapy. After 6 months of treatment, he, remained positive for HCV RNA. These treatments were not only expensive but also resulted in adverse effects, including stomach burning, loss of appetite, nausea, fever, fatigue, and anxiety.

In 2011, Patient was advised to undergo PEG-IFN plus RBV combination therapy. However, he remained a non-responder. After one and a half year, in 2013, patient received the same (PEG-IFN plus RBV) combination therapy for 6 months. Yet patient’s serum was still positive for HCV RNA. Eventually, in 2015, he underwent SOF (400 mg/day) plus RBV combination therapy for 6 months. Still SVR was not achieved and surprisingly a high viral load of 5.2 × 10^5^ IU/ml was reported by real-time polymerase chain reaction (PCR) diagnosis. An ultrasound revealed that his liver was of normal shape, size, and echotexture; he had a mildly fatty liver with no fibrosis or lesion.

The patient’s diagnostic and treatment history are summarized in Tables 1 and 2, respectively. Viral genotype remained undetermined/untypable for the years 2012, 2014, and early 2016 (Table 1) following 6 months of combination therapy (Table 2), perhaps due to the detection method’s incapability or the detection limit as it was performed on conventional PCR-based method followed by detection on the agarose gel. In late 2016, the viral load was found to be 5.2 × 10^5^ IU/ml and the patient was found positive for genotype 3a (Table 1).

**Table 1:** Patient diagnostic history

Diagnostic tests	Method	Results	Date/Year
HCV	Qualitative PCR^a^	Detectable RNA	2003
HCV	Qualitative PCR	Detectable RNA	12–03-2008
Ultrasound	Ultrasound (liver)	Liver is normal shape, size and echotexture, mild fatty liver	2009
HCV	Qualitative PCR	Detectable RNA	06–05-2011
HCV	Qualitative PCR	Detectable RNA	12–12-2011
Ultrasound	Ultrasound (liver)	Liver is normal size, shape and echotexture, no focal lesion, mild fatty liver	23–12-2011
HCV	Qualitative PCR	Non-Detectable	18–04-2012
HCV	PCR Genotyping	Un-typable	24–4- 2012
Liver enzymes	ALT (NR 10–50)	40 IU	8–7-2013
HCV	Quantitative PCR	Detectable RNA 77874 IU/ml	21–5-2014
HCV	PCR Genotyping	Un-typable	27–6-2014
Liver enzymes	ALT (NR 10–50)	36 IU	18–09-2015
Liver enzymes	ALT (NR 10–50)	27 IU	14–12-2015
HCV	Qualitative PCR	Detectable RNA	23–4-2016
Ultrasound	Ultrasound (liver)	Liver is normal size, shape and echotexture, no focal lesion	8–05–2016
HCV	PCR Genotyping	Untypable	28–5-2016
HCV	PCR Genotyping	3a	11–8-2016
HCV	Quantitative PCR	Detectable RNA 5.2 × 10^5^ IU/ml	11–8-2016

*Abbreviations: HCV* hepatitis C virus, *PCR* polymerase chain reaction, *ALT* alanine aminotransferase, *NR* normal range

^a^There was no trend of prescribing quantitative PCR before treatment in Pakistan

**Table 2:** Patient treatment history

Therapy used	Mode of treatment	Start date	End date	Duration of therapy	Result	Side effects/complications
interferon alpha-2b plus RBV	Interferon	9–07-2007	5–01-2008	6 months	Resistant to treatment/Non responder	Stomach burning, Loss of appetite, Nausea, Fever, Fatigue, feeling anxious or aggressive
interferon alpha-2b plus RBV	Interferon	11–07-2010	5–01-2011	6 months	Resistant to treatment/Non responder	Stomach burning, Loss of appetite, Nausea, Fever, Fatigue, feeling anxious or aggressive
PEG-Interferon alpha-2b plus RBV	Interferon	24–08-2011	20–02-2012	6 months	Resistant to treatment/Non responder	Stomach burning, Loss of appetite, Nausea, Fever, Fatigue, feeling anxious or aggressive
PEG-interferon alpha-2b plus RBV	Interferon	11–08-2013	08–02-2014	6 months	Resistant to treatment/Non responder	Stomach burning, Loss of appetite, Nausea, Fever, Fatigue, feeling anxious or aggressive
Sofobuvir plus RBV	Polymerase inhibitor	1–08-2015	1–02-2016	6 months	Resistant to treatment/Non responder	Loss of appetite

After partial genome sequencing of NS5B, BLAST analysis showed 93% similarity to the already existing NS5B nucleotide sequences in the GenBank database (Fig. 2). This shows virus (accession number KY971494; variant ‘Pk1-RV’) is of genotype 3a. Analysis further confirmed that the variant (Pk1-RV) is distinct from HCV genotypes 3 k, 3b, 1a, and 1b (Fig. 2).

**Fig. 1:**
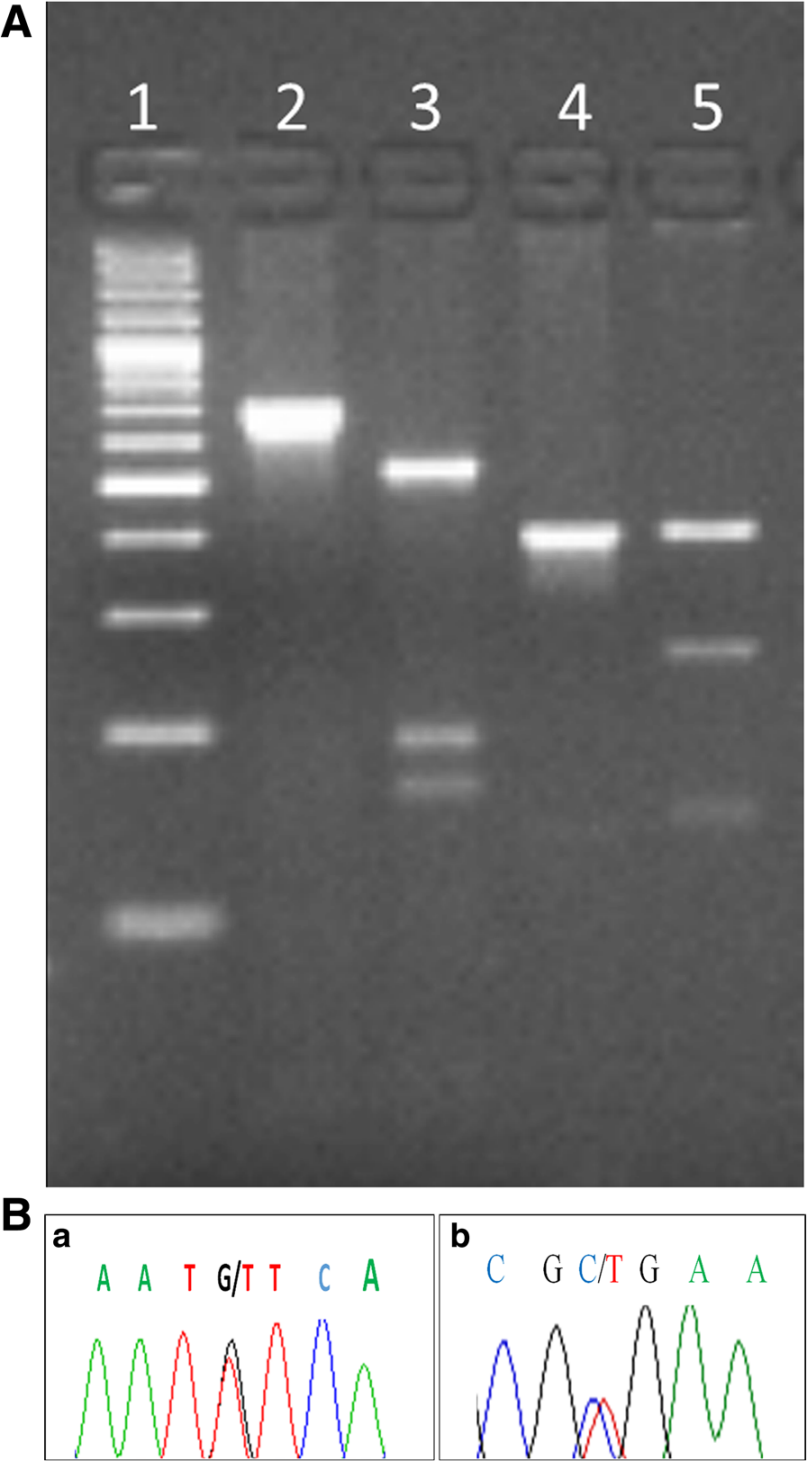
**a** IL-28B polymorphism showing host genotype CT (rs12978960) and TG (rs8099917) in the patient. Line 1: DNA ladder; Line 2: Amplified uncut product of rs12978960; Line 3: CT genotype by RFLP; Line 4: Amplified uncut product of rs8099917; Line 5: GT genotype by Restriction Fragment Length Polymorphism (RFLP). **b** Chromatogram showing (**a**) heterozygous G/T (rs8099917) and (**b**) heterozygous C/T (rs12979860) polymorphism of IL28b

Taking into account the medical history of the patient, we performed restriction fragment length polymorphism (RFLP) for interleukin 28B (IL28B) at rs8099917 and rs12979860. The present study showed polymorphism cytosine and thymine (CT) and guanine and thymine (GT) at (rs12978960, (rs8099917) respectively, as in (Fig. 1a and b). Same polymorphism was reported by Yang et al. who has linked it with successful treatment outcome (SVR) [19].

## Materials and methods

### Patient history and blood sample collection

Patient’s medical record showed that he remained non-responding to multiple antiviral therapies (i.e. IFN + RBV and PEG-IFN + RBV) during 2003–2015 (as stated in Tables 1 and 2). In 2016, patient was found also non-responsive to sovaldi + RBV combination therapy. His blood sample was analysed for viral and host factors such as viral genotyping, viral load, IL28B polymorphism and partial genome sequencing of NS5B.

### Biochemical test

Blood level of liver functional enzyme alanine aminotransferase (ALT) was analysed using the Abcam® Alanine Transaminase Activity Assay Kit (ab105134), (Cambridge, UK) as according to the manufacturer’s instructions.

### RNA and DNA extraction

For viral load determination, genotyping and partial genome sequencing of NS5B, viral RNA was isolated using viral RNA extraction kit (Instant Virus RNA Kit, AJ Roboscreen). The human genomic DNA for IL28B typing is extracted (Phenol chloroform method).

### Viral load

The viral load was determined using a previously reported method [20] through real-time PCR machine (Rotor-Gene 3 000™, Corbett Research, Sydney, Australia). The lower limit of detection of the assay was set at 50 IU/ml [21].

### HCV genotyping

Extracted viral RNA was processed for viral genotyping using a method previously reported by Aziz et al. [22].

### IL28B typing

Polymorphism in the IL28B gene (rs8099917, rs12979860) was detected by amplifying genomic DNA using the primer sets, as described previously [23, 24]. Restriction digestion was performed using BseMI (BsrDI) and Hpy166II and DNA fragments were detected on 2% agarose gel. The gel image pattern confirmed GT and CT genotypes for IL28B (rs8099917 and rs12979860, respectively).

### Phylogenetic analysis of HCV isolate obtained from target patient

Sequencing-based viral genotyping was assessed using NS5B genome sequencing, as reported previously by Ali et al. [25]. Sequencing results showed that the genotype was 3a.

MEGA 7 software [26] was used for assessing the phylogenetic relationship of the partial genome sequence (Pk1-RV; GenBank accession no. KY971494), with the reference sequences obtained from GenBank. HCV NS5B reference sequences from different regions of Pakistan and Tajikistan were compared with the partial viral genome sequence obtained from the patient. A few reference (genotype 1b obtained from GenBank database) sequences were from Tajikistan.

**Fig. 2:**
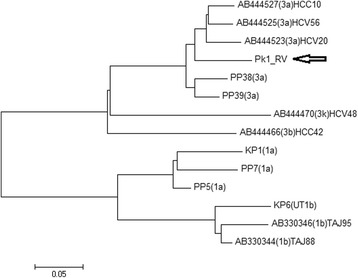
Nucleotide sequence-based genotyping of HCV. The resistant variant is labeled as Pk1-RV (GenBank accession no. KY971494) and clustered with genotype 3a reference sequences. The fragment length of target sequence is 353 nucleotides of NS5B gene. Analysis was carried out using 14 reference viral genome sequences. Sequences included genotype 3a (accession no. AB44523, AB44525, AB44527, PP3, PP39, and KPK2); 3b (accession no. AB444466); 3 k (accession no. AB444470); 1a (KPK1, PP5, PP7); and 1b (KPK6, accession no. AB330344, AB330346)

The resistant variant clustered with out-group, which need further research to get in site of the phenomenon. These sequences include different genotypes and subtypes (3a, 3b, and 3 k, and 1a and 1b). Evolutionary history was inferred using the neighbour-joining method [27]. The optimal tree with the sum of branch length = 1.30808452 was constructed. The percentage of replicate trees in which the associated taxa clustered together in the bootstrap test (500 replicates) are shown next to the branches [28]. The tree was constructed using a neighbor-joining algorithm (Fig. 2).

## Discussion

In present study, we reported a multidrug-resistant HCV-infected patient with genotype 3a and its association with IL28B polymorphism. Ultrasound examination showed that patient’s liver was normal in size, shape and echo texture with no focal lesion. In spite of having favourable virus and host genetic factor i.e. IL28B polymorphism CT and GT at rs12978960 and rs8099917, respectively, and ALT in normal range, we observed higher viremia in blood at the end of sovaldi plus ribavirin treatment.

Sezaki et al. conducted a study to determine the impact of IL28B polymorphism on SVR in patients on a ledipasvir/SOF regimen and observed that genotypes GT and CT were associated with lower response rates [29]. It has been reported that polymorphism in IL28B (rs12979860) is strongly associated with the response rate to the antiviral treatment regimen. The most favourable polymorphism in terms of responsiveness to therapy is cytosine-cytosine (CC) (rs12979860), which has been reported to have an almost two-fold higher likelihood of attaining a SVR as compared to patients with a cytosine-thymine (CT) or thymine-thymine (TT) genotype at the same locus [30].

Previous studies have shown that the TT (rs8099917) and CC (rs12978960) host-genotype population is associated with higher SVR rates as a result of antiviral therapies [31–33]. However, a detailed study needs to be performed to investigate host genotypes in HCV-infected individuals in Pakistan to better understand the pattern of viral response to antiviral therapies.

In the current study, the patient tolerated the SOF and RBV combination therapy well, however, he failed to achieve SVR. Ali et al. recently conducted a systematic review of treatment response rates in Pakistani HCV-infected patients and concluded that IFN plus RBV combination therapies have SVR rates of 64.38–68.38% [31]. Oral treatment against all HCV genotypes, in the majority of patients, is now possible due to the availability of a number of highly effective IFN-free regimens [34]. Kowdley et al. performed a multicenter study and reported that SOF was relatively safe, with rare viral breakthrough during treatment and fewer drug interactions [15]. Akhter et al. conducted the first study in Pakistan that reported SVR rates of 85.5% to RBV plus SOF combination therapy (*n* = 502; genotype 3a) [17]. Patients infected with HCV-3 have been reported to have lower response rates to SOF and RBV therapy than those infected with genotype 2 [16]. Therefore, a combined therapy of RBV and SOF for 24 weeks is an effective treatment regimen for post-transplantation HCV infection [35].

Mutations associated with resistance to DAAs especially SOF have been reported in several studies [36, 37]. However, there is limited information from Pakistan about the selection of DAA-resistant viral mutations [23]. Viral sequences from Pakistan need to be investigated for mutations and/or amino acid substitutions associated with possible non-responsiveness to/relapse associated with antiviral therapies.

## Conclusions

In this case, viral genetic factor (genotype 3a) and host genetic factors (CT at locus rs12978960 and GT at rs8099917) were mildly favourable in terms of responsiveness to therapies. The phylogenetic analysis showed a distinct 3a genotype, which cluster with the 3a genotype from Pakistan however, it was observed that the sequence is more evolutionary diverse on the basis of an increased branched length of the node in the phylogenetic tree. Therefore, it is concluded that mutation in the viral genome and host genetic factor could be responsible for the patient’s non-responsiveness to the therapies. We recommend administration of triple therapies (RBV + INF + SOF/ Boceprevir or RBV + BRF + SOF), which could be effective in non-responding patients. It is also suggested to use a combined therapy of Olysio™ and SOF*.* In addition, further studies should be conducted to understand the possible mechanism(s) of viral non-responsiveness to therapy regimens, with a special focus on Pakistani patients.

## Additional file

Additional file 1: Multilingual abstract in the five official working languages of the United Nations. (PDF 460 kb)

## Abbreviations

BRF: Boceprevir
CT: Cytosine and thymine
DAA: Direct-acting antiviral
GT: Guanine and thymine
HCV: Hepatitis C Virus
IFN: Interferon
IL28B: Interleukin 28B
PCR: Polymerase chain reaction
PEG-IFN: Pegylated interferon
RBV: Ribavirin
RFLP: Restriction Fragment Length Polymorphism
RNA: Ribonucleic acid
SOF: Sofosbuvir
SVR: Sustained virological response
