# Supplementary material for: A case of a patient infected with a hepatitis C virus genotype 3a multidrug resistant variant in Pakistan
Source: Infect Dis Poverty. 2018 Feb 11;7:11. doi: 10.1186/s40249-018-0386-7 (PMC6389057; doi:10.1186/s40249-018-0386-7)
Supplement: Supplementary file 1 — Multilingual abstract in the five official working languages of the United Nations. (PDF 460 kb) [file 40249_2018_386_MOESM1_ESM.pdf]

Translation of the abstract into the five official working languages of the United Nations

حالة مريض بالتهاب الكبد الوبائي سي من النمط الجيني 3أ مقاوم للعلاج المتعدد العقاقير في منطقة باكستانية فقيرة

أسد زياد، محمد علي، حفصة عزيز، محمد ضيا، ظابطة خان شينواري وعبيدة رضا

الخلاصة
نبذة: يعاني ما يقارب عشرة ملايين شخص في باكستان من التهاب الكبد الفيروسي الوبائي (HCV). حيث يصاب معظم المرضى بالالتهاب الكبدي المزمن مع حالات نادرة من الشفاء التلقائي. ورغم ذلك فهناك القليل من المعلومات حول الحالات المقاومة للعلاج المتعدد العقاقير في باكستان.
النتائج: تصف هذه الدراسة حالة مريض ذكر في السابعة والأربعين من عمره شُخِّص بالتهاب كبدي وبائي مزمن من النمط الجيني 3أ في العام 2003. لم يتلق المريض أي علاج بعد التشخيص الأولي لمدة خمس سنوات. بعدها تلقى دورتي علاج من الإنترفيرون (IFN) إضافة إلى ريبافيرين (RBV) عام 2007 و2010، وبالرغم من ذلك لم يستجب للعلاج. تلقى المريض بعدها دورتي علاج إضافيتين من الإنترفيرون المعزز (IFN α-2b) إضافة إلى (RBV) (في عامي 2011 و2013)؛ ولم يستجب للعلاج أيضا.
في عام 2016 خضع المريض للعلاج باستخدام تركيبة من سوفوسبوفير إضافة إلى ريبافيرين وعلى الرغم من ذلك لم يحقق أي تقدم في علاج الفيروس. تبين أن العامل الجيني المضيف متغير لواقح الجوانين والثايمين وكان النمط الجيني لكل من السايتوسين والثايمين (rs8099917) و(rs12979860) بتعدد أشكال صبغي (IL28b) على التوالي. ويشير تحليلنا التطوري أن هذه الحالة المقاومة غير معرفة وقد تتطلب علاجا ثلاثيا.
الاستنتاجات: هذه أول حالة يبلغ عنها بالإصابة بالتهاب كبد فيروسي سي ولا تستجيب للعلاجات المختلفة في باكستان. يجب إجراء دراسات أخرى لفهم الحالات المصابة بالالتهاب الكبدي الوبائي سي والمقاومة للعلاج متعدد العقاقير بين سكان باكستان.

Translated from English version into Arabic by Nancy Muhammed, through

TRANSLATORS
WITHOUT BORDERS

巴基斯坦丙型肝炎病毒基因型 3a 多重耐药变体感染：病例报告

Asad Zia, Muhammad Ali, Hafsa Aziz, Muhammad Zia, Zabta Khan Shinwariand Abida Raza

摘要
引言：巴基斯坦近有一千万人感染丙型肝炎病毒（HCV）。大多数患者罹患慢性肝炎，鲜有自愈。然而，对多重耐药性病毒变异的研究却较少。
病例研究：本病例患者：男性，47 岁，2003 年被诊断出患有慢性 HCV 基因型 3a 感染。初诊后，该患者接受了五年的基础治疗。2007 年和 2010 年，该患者分别接受了两个疗程的干扰素（IFN）和利巴韦林（RBV）治疗，却不见疗效。此后，该患者又接受两个疗程的聚乙二醇化 IFN α-2b 和 RBV 复合治疗（2011 年和 2013 年），然而，也未获得持续的病毒应答。2016 年，该患者接受索非布韦加 RBV 联合治疗，但仍未获得持续的病毒学应答。宿主遗传因子分别在 IL28b 多态性位点 rs8099917 和 rs12979860 上是 GT 和 CT 异质性的。我们的系统发育分析表明，耐药变异属于外出组，可能需要三联疗法。
结论：在巴基斯坦，这是关于 HCV 感染者对多种疗法均无反应的第一例病例报告。基于此，需要进一步的研究来深入了解巴基斯坦人群中多重耐药 HCV 变异体。

Translated from English version into Chinese by Jin Chen, through

CDC
CHINESE CENTER FOR DISEASE CONTROL AND PREVENTION
NATIONAL INSTITUTE OF PARASITIC DISEASES

Un cas d’infection par un variant du virus de l’hépatite C de génotype 3a multirésistant dans une zone défavorisée au Pakistan

Asad Zia, Muhammad Ali, Hafsa Aziz, Muhammad Zia, Zabta Khan Shinwariand Abida Raza

Résumé
Contexte : Quelque 10 millions de personnes sont infectées par le virus de l'hépatite C (VHC) au Pakistan. La plupart développent une hépatite chronique, avec quelques rares cas d'élimination spontanée du virus. On sait cependant peu de chose à propos des variants viraux multirésistants dans le pays.
Observations : Cette étude de cas décrit un patient âgé de 47 ans, chez lequel une infection chronique par un VCH du génotype 3a a été diagnostiquée en 2003. Après le diagnostic initial d'infection virale, le patient est resté sans traitement pendant cinq ans. Il a reçu deux cycles d'interféron (IFN) et de ribavirine (RBV) en 2007 et 2010, sans répondre à ces traitements, puis deux cycles d'IFN α-2b pégylé et de RBV (en 2011 et 2013), toujours sans réponse. En 2016, il a été traité avec du sofosbuvir et de la ribavirine, sans que l'on obtienne encore une réponse virologique. Le facteur génétique de l'hôte s'est avéré résider dans des génotypes hétérozygotes guanine-thymine (GT) et cytosine-thymine (CT) pour rs8099917 et un polymorphisme de rs12979860 dans IL28b, respectivement. Notre analyse phylogénétique suggère que le variant résistant appartient à un groupe à part et pourrait nécessiter une trithérapie.
Conclusions : Ceci est le premier rapport de cas d'un patient infecté par le VIH ne répondant pas à plusieurs traitements au Pakistan. D'autres études sont nécessaires pour comprendre les variants multirésistants du VHC dans la population pakistanaise.

Translated from English version into French by Suzanne Assenat, through

TRANSLATORS
WITHOUT BORDERS

Описание клинического случая пациента из бедных городских кварталов Пакистана, инфицированного мультирезистентным вариантом вируса гепатита С генотипа 3а

Асад Зиа/Asad Zia, Мухаммад Али/Muhammad Ali, Хафза Азиз/Hafsa Aziz, Мухаммад Зиа/Muhammad Zia, Забта Хан Шинвари/Zabta Khan Shinwari и Абида Раза/Abida Raza

Краткое изложение
Исходная информация: Около 10 миллионов людей в Пакистане инфицированы вирусом гепатита С (HCV). У большей части пациентов гепатит перешел в хроническую стадию, в редких случаях наблюдался спонтанный клиренс. Однако, имеется очень мало данных о мультирезистентном варианте вируса в Пакистане.
Полученные результаты: Данное исследование клинического случая описывает заболевание 47-летнего мужчины, у которого в 2003 году был диагностирован хронический вирус гепатита С генотипа 3а. После постановки первичного диагноза «вирусная инфекция» пациент не получал предварительного лечения на протяжении пяти лет. В 2007 и 2010 гг. пациент прошел два цикла терапии интерфероном (IFN) в сочетании с рибавирином (RBV), но у пациента наблюдалась резистентность к лечению. Затем пациент прошел два дополнительных терапевтических цикла лечения пегилированным интерфероном α-2b в сочетании с рибавирином (в 2011 и 2013); реакции на лечение по-прежнему не было. В 2016 пациент прошел комбинированную терапию софосбувиром в сочетании с рибавирином, однако устойчивый вирусный ответ все еще не был получен. Выяснили, что генетическим фактором носителя являются гетерозиготные генотипы гуанина и тимина (GT) и цитозина и тимина (CT) в результате полиморфизма IL28b (rs8099917 и rs12979860, соответственно). Проведенный нами филогенетический анализ позволяет предположить, что резистентный вариант относится к внешней группе, и может потребоваться трехкомпонентная терапия.
Выводы: Данный клинический случай является первым случаем в Пакистане, в котором сообщается о пациенте, инфицированном вирусом гепатита С и не отвечающий при этом на лечение с применением нескольких препаратов. Необходимо проведение дальнейших исследований, чтобы разобраться в мультирезистентных вариантах вируса гепатита С у

пациентов пакистанского происхождения.

Translated from English version into Russian by Helga_Sh, through

Un caso de paciente infectado con una variante del genotipo 3a del virus de hepatitis C resistente a varios medicamentos en una zona desfavorecida de Pakistán

Asad Zia, Muhammad Ali, Hafsa Aziz, Muhammad Zia, Zabta Khan Shinwariand Abida Raza

Resumen

Información de referencia: En Pakistán hay alrededor de 10 millones de personas infectadas con el virus de la hepatitis C (HCV). La mayoría de los pacientes desarrolla hepatitis crónica y solo raramente desaparece de forma espontánea. Sin embargo, se sabe poco sobre las variantes del virus resistentes a varios medicamentos en Pakistán.

Resultados: En el estudio de este caso se describe a un varón de 47 años al que se ha diagnosticado infección por genotipo 3a de HCV crónico en 2003. Tras el diagnóstico inicial de la infección por el virus, el paciente no recibió tratamiento durante cinco años. Posteriormente recibió dos ciclos de terapia con interferón (IFN) y ribavirina (RBV) en 2007 y 2010, sin que respondiera a la terapia. El paciente recibió entonces dos ciclos de tratamiento con IFN α-2b pegilado y RBV (en 2011 y 2013), también sin respuesta. En 2016, el paciente fue sometido a una terapia combinada de sofosbuvir y RBV, pese a lo cual no se consiguió una respuesta viral sostenida. Se averiguó que el factor genético del portador correspondía a los genotipos heterocigóticos de guanina y timina (GT) y citosina y timina (CT) con polimorfismo rs8099917 y rs12979860 de IL28b, respectivamente. Nuestro análisis filogenético sugiere que la variante resistente pertenece a un grupo extraño y puede requerir triple terapia.

Conclusiones: Éste es el primer caso que informa sobre una persona infectada de HCV que no ha respondido a varias terapias en Pakistán. Es preciso realizar más estudios para conocer las variantes del HCV resistentes a varios medicamentos entre la población de Pakistán.

Translated from English version into Spanish by SergioLorenzi, through
